# Feasibility of couple-based expanded carrier screening offered by general practitioners

**DOI:** 10.1038/s41431-019-0351-3

**Published:** 2019-02-11

**Authors:** Juliette Schuurmans, Erwin Birnie, Lieke M. van den Heuvel, Mirjam Plantinga, Anneke Lucassen, Dorina M. van der Kolk, Kristin M. Abbott, Adelita V. Ranchor, Agnes D. Diemers, Irene M. van Langen

**Affiliations:** 1 Department of Genetics, University of Groningen, University Medical Center Groningen, PO Box 30.001, 9700 RB Groningen, The Netherlands; 2 Clinical Ethics and Law, Faculty of Medicine, University of Southampton, Tremona Road, SO16 5YA Southampton, UK; 3 Department of Health Psychology, University of Groningen, University Medical Center Groningen, PO Box 30.001, 9700 RB Groningen, The Netherlands; 4 Department of General Practice and Elderly Care, University of Groningen, University Medical Center Groningen, PO Box 30.001, 9700 RB Groningen, The Netherlands

**Keywords:** Ethics, Genetic counselling, Genetic testing, Human behaviour

## Abstract

Expanded carrier screening (ECS) aims to inform couples’ reproductive choice, preferably before conception. As part of an implementation study in which trained general practitioners (GPs) offered a population-based ECS couple-test, we evaluated the feasibility of the test-offer and degree of participant informed choice (IC). Trained GPs from nine practices in the northern Netherlands invited 4295 female patients aged 18–40 to take part in couple-based ECS. Inclusion criteria were having a male partner, planning for children and not being pregnant. We evaluated the feasibility of the organizational aspects, GP competence and the content of the pre-test counselling. Participant satisfaction, evaluation of pre-test counselling and degree of IC were measured using a longitudinal survey. We explored GP experiences and their views on future implementation through semi-structured interviews. 130 consultations took place. All participating GPs were assessed by genetic professionals to be competent to conduct pre-test counselling. Most (63/108 (58%)) consultations took place within the planned 20 min (median 20, IQR 18–28). GPs considered couples’ prior knowledge level an important determinant of consultation length. 91% of patients were (very) satisfied with the GP counselling. After pre-test counselling, 231/237(97%) participants had sufficient knowledge and 206/231(88%) had a positive attitude and proceeded with testing. Our pilot demonstrates that offering couple-based ECS through trained and motivated GPs is feasible. Future large-scale implementation requires a well-informed general public and a discussion about appropriate reimbursement for GPs and health care coverage for couples. Providing (more) test information pre-appointment may help reduce average consultation time.

## Introduction

Next generation sequencing enables simultaneous screening for carrier status of many genes associated with autosomal recessive (AR) conditions and some X-linked conditions, called expanded carrier screening (ECS) [1]. Where ECS is done prior to pregnancy, couples found to be at increased risk of having a child affected by such a condition can consider alternative reproductive options such as in vitro fertilization with pre-implantation genetic diagnosis (PGT-M) or prenatal testing (PND) (with possible termination of an affected pregnancy). However, a population-based ECS is not yet part of regular pre-conception care in public health care systems, but several private companies and some academic centers have started to develop and offer ECS tests for individuals or couples planning to conceive [2]. ECS aims to inform a couple about their risk of conceiving children with these genetic conditions. In this paper, describing the first population-based implementation pilot of an ECS test-offer by GPs, we decided to focus on severe AR conditions.

The Genetics Department of the University Medical Centre Groningen (UMCG) in the Netherlands has developed and validated a couple-based ECS test for 50 AR conditions associated with approximately 70 genes [3]. These conditions were selected because they are early onset, serious diseases that result in severe physical or intellectual disabilities, severe pain, or premature death. These criteria were recommended by an international expert meeting at the UMCG in 2013, are supported by literature [4–6] and current guidelines which include criteria related to severity of illness [1]. In the Dutch population approximately 1 in 150 couples are carriers for the same condition in this test [7]. For severe AR conditions, the risk of being a carrier couple for an AR condition is about 1% [8]. The percentage of at risk couples that can be identified through ECS in the general population depends on the composition of the test-panel. For example, when 500 conditions (which are not all serious) are included, detection rate is higher [9] than in our (conservative) panel. Given that being a carrier of *any* AR condition is common but the chance of carrying a particular condition is very low, it is the positive combined ‘couple-result’ which conveys clinical utility for reproduction. We therefore argue that a responsible approach to implementing ECS in a public health care system is to offer it as a couple test and provide couple results only. This approach is supported by the recently published Belgian guidelines [10]. In the test results, we report only causal recessive variants, including known deleterious variants listed in databases (e.g., Human Gene Mutation Database, Biobase, Qiagen), and variants predicted to truncate or affect gene expression.

We have previously reported that both health care professionals (HCPs) and the target population support couple-based ECS in the general population with the GP as preferred provider [3, 11], and other studies have demonstrated that carrier testing for single-gene disorders such as cystic fibrosis (CF) and hemoglobinopathies in primary care is feasible and acceptable [12, 13]. More than 99% of the Dutch population are registered with a GP [14], and most GP care is included in the mandatory health insurance package all Dutch citizens carry. In the Dutch healthcare system, GPs play a central role as gatekeeper for secondary or tertiary care [15], which makes extending their current preconception care responsibilities to include a population-based ECS offer a logical approach. We therefore investigated whether test-provision by GPs could be a feasible approach for ECS and result in informed choice of couples who attended pre-test counselling. As most general HCPs lack the skills, confidence and knowledge to communicate clinical genetics issues [1, 16–18], we designed and provided training to GPs and subsequently performed an implementation study where these trained GPs offered the UMCG ECS test to couples from the general population. This current study is part of a larger study on the feasibility, uptake, and psychological impact of the test-offer.

## Methods

### Provision of ECS test-offer and care

**Fig. 1:**
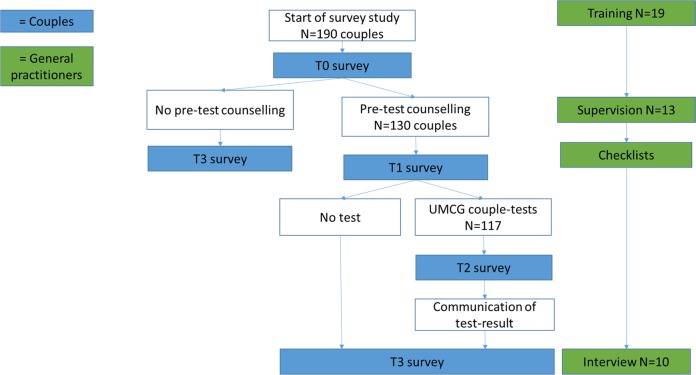
Overview of ECS test-offer, provision of care and study design.GPs provided pre-test counselling to couples interested in ECS testing. Subsequently, couples could decide to proceed with testing. We used a mixed-methods longitudinal study in which assessments were made at four time points (T0-T3) through either questionnaires and/or semi-structured interviews, with couples and participating GPs as study participants

Fig. 1 displays the ECS test-offer and provision of care. By sending out an invitation letter, participating GPs offered all potentially eligible women registered in their practices the opportunity to take part in the ECS testing program. Couples who were interested in the ECS test could make an appointment for pre-test counselling with the inviting GP. Afterwards they would decide whether or not to proceed with testing. The ECS-test was only accessible to couples who received pre-test counselling and couples were required to attend pre-test counselling together.

Trained GPs were asked to provide pre-test counselling about ECS in combination with general preconception care (GPC) advice (e.g., advice about folic acid supplementation, cessation of alcohol use or smoking). For this counselling, a double-consultation time was available (20 min). Referral to the Clinical Genetics department of the UMCG was available for couples identified to have prior increased risk, e.g., due to suspected family history of a genetic condition. Couples who proceeded with the ECS test could give a blood sample using request forms provided by the GP. The UMCG Genomics Laboratory performed the test. With a turn-around time of 8 weeks, GPs received a result for couples who provided blood samples and then communicated the results to them. A couple was considered a carrier couple if *both* couple members have a class IV or V variant in one of the recessive disease genes included in the test.

Carrier couples, but also non-carrier couples with remaining questions, could be referred to Clinical Genetics for post-test counselling. We also launched a publicly accessible website with general information about the 50 AR conditions, the ECS test, and related procedures (www.dragerschapstest.umcg.nl). The research team, including the genetic counsellor involved, could be contacted through the website. There were no patient expenditures associated with the study and test-participation. PGT-M and PND for serious conditions such as those included in this ECS-test are available to high risk couples. In the Netherlands, costs of PGT-M and prenatal testing are covered by statutory health insurance.

### Study design

Figure 1 also depicts the study design. We used a mixed methods longitudinal design with four study time points (T0–T3), with couples and GPs as study participants. GPs were asked to evaluate each individual pre-test counselling at T1. At T3 they were invited to take part in semi-structured one-to-one interviews to explore their overall experience with test-provision. A genetics professional involved in the training supervised the first two pre-test counselling sessions of each GP. Couples were asked to fill out an online survey. Couples who attended pre-test counselling received three (T0, T1, T3) to four (T0, T1, T2, T3) questionnaires, depending on whether they proceeded with testing. The study protocol was approved by the UMCG Medical Research Ethics Committee (METc 2015/384).

### Recruitment of GPs

Study participation was open to GPs in the catchment area of the UMCG. Staff from the Genetics Department and the Department of General Practice first approached potentially interested GPs personally and a recruitment message was added to a newsletter for GPs. GPs from 34 practices received the study information. Nineteen GPs from nine practices agreed to participate. One practice (no. 9) withdrew during the study because they were too busy to facilitate study participation. No further invitations were sent, but participants already included could still attend pre-test counselling, and proceed with testing.

Prior to the start of the study, all GPs were required to participate in a 2.5 h training session about pre-test counselling developed by the research team. This training session included background information about ECS and other general aspects of preconception care and an interactive session about reproductive genetic pre-test counselling. An information booklet was provided to complement the training that provided background information and a counselling guideline with important items to discuss. Two weeks after the training, all GPs filled out an online questionnaire testing their knowledge. GPs received individualized feedback on their incorrect answers prior to start of the counselling. Support from the clinical genetics professionals was available as needed throughout the study.

### Recruitment of couples

Between January and December 2016, participating GPs selected and invited all potentially eligible women aged 18-40 registered in their practices to participate. Eligibility criteria were not being pregnant, having a (male) partner and planning to have children with this partner. Pregnant women were excluded, because the turnaround time of the test-result was a maximum of eight weeks at that time. Additionally, the extra skills probably needed for ‘urgency counselling regarding ECS in pregnancy’ requires different counselling skills of GPs which were not yet part of our preparatory training. The women were asked to invite their partners in the study. Couples who were interested in the ECS test could make an appointment for pre-test counselling with the GP after both partners had given written consent to participation. They could decide whether to proceed with testing after attending this appointment. Invitations were sent by mail and included a letter signed by the GP, a response card and an information leaflet. This test information leaflet consisted of the type of conditions included in the test, the chances of being a carrier couple and of having an affected child, reproductive options available for carrier couples, and test procedures. It also included a link to the website for further information. Women who were eligible and interested in participating received more detailed study information.

### Measures

Feasibility was evaluated in terms of the organizational aspects of this GP-provided ECS test-offer and the provision of care, with a focus on the pre-test counselling. We explored GP experiences and views on the ECS test to evaluate feasibility and improve future implementation. We adopted Marteau et al., (2001)’s definition of IC, who developed the Multidimensional Measure of Informed Choice (MMIC) to measure IC in relation to prenatal screening for Down’s syndrome [19]. A choice was considered 'informed' if participants had sufficient knowledge and accepted the test offer (in case of a positive attitude) or declined the test-offer (in case of a negative attitude) [19]. Table 1 displays the topics and items investigated, which are based on relevant literature [1, 16, 17, 20–22]. The organizational aspects of the ECS test-offer were evaluated quantitatively as the time used for pre-test counselling and qualitatively through analysis focused on barriers and facilitators. Pre-test counselling was evaluated in terms of competence, content and patient-satisfaction. Competence was judged by the genetics professionals after supervision and evaluated by GPs during the interviews. Both couples and GPs evaluated the content. Couples also rated their satisfaction with pre-test counselling. Specific measures, instruments and details are described in Suppl. 1.

**Table 1:** Overview of items used to measure feasibility and informed choice

Topics	Quantitative		Qualitative
	Instrument, time point (subject)	Items/measures	
A. Feasibility			
1. Organizational aspects of GP-provided ECS test offer	Checklist at T1 (GP)	Start and end time of pre-test counselling sessions	Barriers and facilitators of:
			• Duration of pre-test counseling
			• Both partners attending pre-test counselling
			• Communicating test-result
			• Referrals
2. Evaluation of care: competence and satisfaction	Survey at T1 (couples)	Patient satisfaction (overall + CGSI(1), see supplementary materials	• Self-judgment GPs during interviews
			• Professional judgment genetics professional after supervision
3. Evaluation of pre-test counselling: content	Checklist at T1 (GP)	Items discussed during counselling.	Barriers and facilitators of: Discussing the aspects included on the checklist
	Survey at T1 (couples)	Importance and length of items discussed during counselling.	
4.Views about implementation	Interview at T3 (GP)	N.A.	
B. Informed choice			
Informed choice	Survey at T0 and T1 (couples)	Informed choice measured using adapted MMIC(2), see supplementary materials	

### Quantitative data and analysis

Data on the duration of the consultation and items discussed during pre-test counselling were collected by a checklist for GPs that was filled out after each pre-test counselling (T1). The checklist included eleven items that GPs were required to discuss during pre-test counselling (see supplemental information for list). They were asked to indicate if they discussed the item (yes, somewhat, no), and if not, why not. Data on items discussed during pre-test counselling (i.e., their perceived importance and time spent on them), satisfaction with pre-test counselling and informed choice were collected by couples’ questionnaires using the Roqua online tool for confidential clinical data collection [23]. The IC measure consisted of five knowledge items capturing essential information about ECS testing and two attitude items. We also asked couples to fill out these knowledge items after pre-test counselling as part of our provision of care to verify that couples who proceeded with testing were aware of the correct information. We would call them for additional discussion if they answered any of the five questions incorrectly. Couples could refrain from having the test after this additional information, which happened in one occasion. Data on consultation duration, items discussed during counselling, patient satisfaction, and informed choice were described using percentages, mean (SD) or median (IQR) where appropriate, using SPSS IBM version 23.

### Qualitative data and analysis

Ten semi-structured one-to-one interviews were held with GPs. Two GPs who conducted counselling did not participate due to lack of time and the GP who withdrew from the study did also not participate. A topic guide was developed containing open-ended questions related to the feasibility aspects of this GP-provided test. Interviews were conducted by a trained researcher (JS), audio-recorded and transcribed verbatim. The average duration of the interviews was 41 minutes (range 20-60 min). Data analysis was conducted according to the framework approach of Ritchie and Spencer [24]. Framework analysis follows a process of familiarization, summarizing and coding, which results in matrices presenting the data per theme and case to allow more in depth analysis and comparison across interviewees. Atlas –ti (version.5.2.18 copyright 1993-2018 by ATLAS.ti Scientific Software Development GMbH Berlin) was used to facilitate analysis. Two researchers (JS, LvdH) independently coded the first three interviews, and differences in coding were discussed until consensus was reached. LvdH subsequently coded all interviews, including the first three, while JS coded parts of all interviews randomly and where LvdH had doubts. Final thematic framework matrices were subsequently discussed within the research group until consensus was reached. The preliminary conclusions were returned to the interviewees for member checking [25]. We received six forms, all confirming our conclusions.

## Results

### Inclusion and response

Table 2 shows that 19 GPs attended the training and 130 couples attended pre-test consultation of whom 117 proceeded with testing. Six trained GPs did not conduct pre-test counselling for reasons unrelated to the study. A genetic counsellor conducted one of the pre-test counselling sessions because one couple found out they were already pregnant after they had made their GP appointment. This couple was excluded from the analysis. Ten GPs participated in the interviews. 240/260 (92%) of the individual participants responded to the evaluation of the pre-test counselling. GPs returned 116/129 (90%) checklists.

**Table 2:** Overview of participating GPs, pre-test counselling and tests performed per practice

Participating practice ID (interview no.)	Type of practice	GPs attended training	No. GPs conducted counselling	No. women invited	No. pre-test counselling sessions	No. couple-tests performed
1 (6)	City	1	1	500	24	23
2 (4)	City	1	1	528	12	12
3 (3)	Village	2	1	276	4	3
4 (8&9)	Town	6	4	1045	23	20
5 (2&5)	Town	3	2	780	27	25
6 (1)	Town	1	1	407	18	14
7 (7)	Village	1	1	262	5	5
8 (10)	Town	2	1	330	5	4
9 (NA)	City	2	1	167	12	11
Total		19	13	4295	130	117

### Evaluation of organizational aspects

58% of the pre-test counselling sessions lasted 20 minutes or less, with a median (IQR) of 20 minutes (18–28), indicating that the allocated time of 20 minutes was sufficient for the majority of sessions. Qualitative findings from GP interviews are illustrated with quotes presented in Table 3. Several GPs noted that couples were well informed beforehand, and that this helped them provide counselling within this time. GPs expected pre-test counselling sessions to last longer, if couples were less well-informed, or for couples with little educational background. Some GPs mentioned that over time they developed a routine for conducting the counselling, which reduced the time required for preparation and counselling itself. GPs were positive about attendance of both partners at counselling because the couple-test affects both partners equally and because they considered discussing GPC with both partners important. No carrier couples were identified. GPs did not experience any barriers in communicating the normal results or to referring any couples at normal risk to Clinical Genetics for additional pre- or post-test counselling. GPs or their healthcare assistants communicated the test results by phone, email, or a combination, and some provided the couples with the lab results letter as well.

**Table 3:** GP quotes from interviews

Feasibility aspect	Quote (Interviewee)
Evaluation of care: organizational aspects of the GP-provided test-offer	“I particularly liked the training course, which was essential. It would be difficult to provide the ECS test without doing the training course first.” Interviewee 10
	“At first, I thought 30 minutes should be planned for each consultation… But later I reduced it to 20 minutes, because it was feasible in 20 minutes… Also because at a certain moment you know what to discuss. Well, and people were often perfectly able to tell about the test. Most of them.” Interviewee 5
Evaluation of care: content	“I discussed the items on the checklist with everyone, because I thought those were the essential points. So [amongst others] about what types of diseases were included. What the chances were, that it [the ECS test] does not offer any guarantee [of a healthy baby], and that there were no costs involved [for the couple]. That’s it, in brief.” Interviewee 5
	“What is really important is that they realize that it’s the couple being tested and not the individuals, that the result says nothing about each individual only something about the couple together.” Interviewee 3
Views on future implementation: Suitable provider	[reasons why the GP is suitable]…“well, of course it’s close to the patient, most patients, even these healthy young people know their GP. And that means that, in a counselling like this, the threshold to ask questions is likely to be lower, or to return. They know where to find us when they need to.” Interviewee 8
Views on future implementation	“Well.., I think that with the right provision of information, it could very well be part of this general preconception care advice.” Interviewee 4
Views on future implementation	“The solidarity [healthcare insurance] system here [in the Netherlands] means that if you want to reach people, you should cover the costs.” Interviewee 7

### Evaluation of pre-test counseling

Based on their experiences in this study, GPs and genetics professionals considered training test-providers essential to ensuring quality of the test provision. After GPs were supervised twice, the genetic professionals considered all thirteen GPs competent to conduct counselling on their own. Counselling support from the clinical genetics professionals was requested twice for couples who were pregnant during the study and once for a couple who had misunderstood the purpose of the test. All GPs interviewed said they felt able to provide the pre-test counselling mainly because of the training, supervision and additionally provided materials. Some GPs specifically said they used the study checklist as a practical guidance, and all felt this covered the essential aspects of a pre-test counselling well. Participants evaluated the pre-test counselling with a mean satisfaction score of 4.7/5 (SD 0.5). The majority of participants (54.7%) gave the highest score of 5.0. 91% of participants were *satisfied* or *very satisfied* with GP pre-test counselling.

GPs and couples evaluated the content of the pre-test counselling as follows. GPs indicated that most aspects included on the checklist, apart from GPC and ‘communication and turn-around time of the test-result’, were at least discussed ‘somewhat’ in more than 90% of consultations. Some participants indicated that they thought too little time was spent on discussing the conditions included in the test (55 respondents (23%)) and the follow-up options for high-risk couples (38 respondents (16%)). Some GPs explained they did not discuss each condition in detail, instead discussing the conditions as categories as explained during the training. While GPs indicated that in 36 consultations (31%) they either “did somewhat” or “did not” discuss couples’ reproductive values, more than 85% of participants indicated that the time spent on their and their partners’ values was exactly right.

Most GPs were positive about combining ECS pre-test counselling with GPC. GPs indicated that, for example, due to lack of time, they “did not” discuss GPC in 31% or discussed it “only somewhat” in 14% of consultations. Some GPs explained during the interviews that the counselling might become too complex preventing couples from remembering both. GPC was considered important or very important to discuss by 159 participants (67%), of whom 19/159 (12%) thought too little time was spent on this. In contrast, 167 participants (70%) thought the right amount of time was spent discussing GPC.

### Informed choice

After pre-test counselling by the GP, the number of participants with a sufficient level of knowledge had improved from 195/237 (83%) to 231/237 (97%) (Table 4). Five of six participants who displayed insufficient knowledge –and a positive attitude- after pre-test counselling, proceeded with testing. Another seven participants did not proceed with testing, even though their attitude was positive and knowledge sufficient. Our provision of care pathway –as described in the methods section- prevented participants to make a final decision based on insufficient knowledge.

**Table 4:** informed choice before and after pre-test counselling by the GP

Before pre-test counselling (T0) (*n* = 237)	Positive attitude *n* (%)	Negative attitude *n* (%)	Neutral attitude *n* (%)	Total *n* (%)
Sufficient knowledge	173 (83)	0 (0)	22 (79)	195 (83.)
Insufficient knowledge	36 (17)	0 (0)	6 (21)	42 (18)
Total	209 (88)	0	28 (12)	237
After pre-test counselling (T1) (*n* **=** 237)	Positive attitude *n* (%)	Negative attitude *n* (%)	Neutral attitude *n* (%)	Total *n* (%)
Sufficient knowledge	213 (90)	0 (0)	18 (8)	231 (97)
Insufficient knowledge	5 (2)	0 (0)	1 (0)	6 (2.5)
Total	218 (92)	0 (0)	19 (8)	237

### GP views on future implementation

In line with our previous research, after having offered ECS testing, GPs considered themselves as the most suitable providers for a population-based ECS couple-test. Advantages they mentioned were the low-threshold of GP care, their familiarity with their patients and their background. One GP mentioned that ECS-provision as standard care by all GPs might not be feasible because not all may be able to keep up with technological advances in genetics. Some GPs suggested that only motivated GPs willing to do so should be trained to provide ECS. These GPs could become specialized in (reproductive) genetics, just as some GPs are currently specialized in areas such as palliative or elderly care. Potential barriers that GPs mentioned were resistance to additional workload in already too busy practices or negative attitudes towards ECS. The eight-week turnaround time in our study, was considered acceptable by the GPs for non-pregnant couples. For future implementation, several GPs suggested the laboratory could also send the test result directly to couples. Negotiations with health insurance companies and policy makers were considered necessary to decide on a proper reimbursement fee for test-provision and whether to include ECS in the statutory health insurance package (Table 3).

## Discussion

In this paper we have presented the design of our implementation study of a GP-provided ECS couple-test and our results on its feasibility and the degree of informed choice in couples attending pre-test counselling. Implementing ECS responsibly requires a novel approach [1], and our previous research suggested an important role for GPs [3, 11]. Our study demonstrates that implementing an ECS couple-test consisting of a limited set of severe conditions in the GP setting is a feasible approach that results in an informed decision in most cases.

Importantly, all participating GPs felt and were judged competent to conduct pre-test counselling after being given training, supported by genetic professionals on demand, and assisted by a counselling-checklist. Participating couples were very satisfied with GP pre-test counselling and the Dutch Society of General Practitioners recently stated their support for (more) studies investigating the implementation of ECS in primary care [26]. This approach therefore has the potential to address the concerns about the current lack of genetic literacy and counselling skills among non-genetics HCPs providing genetic tests [1, 18–20], and our results can inform options for responsible mainstreaming in genetics.

Most pre-test counselling sessions were conducted within the allotted time span of 20 minutes, with additional counselling sometimes needed to discuss GPC. In some situations, it might be more effective to separate the two types of counseling: directive (e.g., advice not to smoke or drink alcohol) and non-directive (facilitate reproductive decision-making in line with couples’ values).

A study of CF carrier testing in primary care showed that GPs could conduct the (less complex) counselling in an average of 12 minutes [12]. According to participating GPs, pre-test counselling within the allocated time was facilitated because couples were already well-informed, perhaps due to the extensive study information, website and the questionnaires participants filled out.

Our results suggest that GPs could have extended their pre-test discussion of the reproductive options available for couples who are found to be both carriers of the same condition, which would also include an assessment of the value system held by that couple. Such discussions are standard practice for GPs, but our future training could be adjusted to focus more on these aspects in the preconception setting. Couples do not often request preconception consultations from GPs or other HCPs in the Netherlands [27], thus an added benefit of the ECS test-offer meant that GPs could discuss or follow-up on GPC advice with more couples –and both partners- than was routine. Future research could also explore whether prenatal carrier screening is feasible in this setting and what necessary adjustments should first be made in training and test-delivery. In the study we required both partners to attend pre-test counselling together and GPs agreed that it was preferable to include both partners jointly in the discussion of ECS as this affects both prospective parents. To lower practical barriers to attend counselling, in the future GPs could use web-consultations or face-to-face consultations at times desired (evenings/weekends), although this requires additional training and adjusted infrastructure.

### Considerations regarding large-scale implementation of ECS in primary care

Our research concentrated on the offer of ECS within primary care. Eligible women were actively and individually approached by their GP by letter. Large scale implementation could also be a more passive and collective approach, e.g., via posters, leaflets and information about the test on GPs’ websites. However, this requires the public to become more knowledgeable on this topic, which means more educational efforts would need to be aimed at this group. Moreover, couples could fill out an online decision-aid in advance to inform and prepare them and facilitate efficient and effective pre-test counseling. No major barriers to large-scale implementation were mentioned by GPs in our study provided they can use 20 minutes for the counselling and that there are no financial barriers for them and their patients. Our results should inform discussions with relevant stakeholders to negotiate reimbursement for the consultation as well as the test.

GPs in our study suggested that ECS could be provided by ‘specialized’ GPs who focus on a specific aspect of GP care. Not all GPs may be interested in investing the time and effort necessary to obtain and maintain the required counselling skills, considering that the total number of counselling sessions per GP might be relatively low. The specialization approach would guarantee the necessary minimum number of pre-test counselling sessions per GP per year to maintain competence. GP specialization already exists in the Netherlands in areas such as elderly and palliative care. Other primary HCPs involved in preconception care—such as midwives, community pediatricians or nurse practitioners—might also be willing to offer ECS. In all scenarios, the role of Clinical Genetics in a population-based ECS couple-test could focus on education, support/auditing and post-test counselling for carrier couples.

### A couple-based test for severe recessive conditions only

Salient features of our approach to ECS were the well-considered composition of the test-panel and the provision of couple-only results for this population-based offer through participating GPs. The composition of the panel facilitated a generic type of consent and the couple-based strategy resulted in a minimal need for post-test counselling by the GP or Clinical Genetics professionals. As time to discuss all conditions in detail is limited and some couples desire more information, extensive information about the conditions should be easily accessible for couples, as was the case on our website. Not disclosing individual results remains a matter of debate given the perceived utility for cascade screening [22], as well as the participants’ personal preferences [28].

We argue that previous cascade screening approaches disclosing individual results, e.g., for relatively frequent conditions, are no longer helpful when switching to population-based ECS, especially given that everyone is likely to be a carrier of one or more recessive conditions. If ECS was well known to the public and to HCPs and there were no (financial) barriers to participating, the new approach would be to offer ECS testing to all couples wishing to reproduce. Whilst some have expressed concern that individual results are important if couples split up, our response to this is that a new couple test, i.e., a re-analysis of the couple’s or one of the couple’s stored data, could be done in those cases. In the Dutch health care system these data (and the DNA) are stored for these and other purposes. A referral to Clinical Genetics would only be necessary for couples with prior/suspected increased risk due to family and/or personal health history and/or ethnic background. The approach suggested in this study applies for ECS aimed at AR diseases only. Our couple-based approach for severe AR conditions could (and perhaps should) be complemented with individual screening for more prevalent X-linked conditions like Fragile-X and Duchenne muscular dystrophy. It is important to evaluate in future studies what would be necessary aditionally to facilitate counselling and test-provision and which adjustment would be needed for responsible implementation in the Dutch public health system. In this paper we focused on free couple-based ECS in the Dutch public health system. We anticipate that for non-reimbursed ECS and ECS in a private setting, arguments for couple-based ECS or reporting of individual carrier results could well be different. A discussion of these arguments is beyond the scope of this paper.

### Conclusions and recommendations

This GP-provided couple-based ECS test for a limited number of severe AR conditions in the setting of preconception care, presents a timely and responsible option to inform couples planning a pregnancy about their chances of having a child affected by a severe genetic condition. This approach was not only feasible in our setting, but also led to an informed choice for most participants. Future national implementation could involve other dedicated GPs, or other primary HCPs willing to be trained to provide the test, given that support as well as practical tools from a clinical genetics service are available. Furthermore, some factors identified in our study should be considered, such as raising public awareness to facilitate a well-informed population and resolution of reimbursement issues. Our approach, that was feasible in the (northern) Netherlands, might be transferable to other (European) public health systems with easily accessible primary health providers who are willing to be trained and have the necessary resources to offer ECS.

## Supplementary information

Supplementary materials
