# Supplementary material for: Feasibility of couple-based expanded carrier screening offered by general practitioners
Source: Eur J Hum Genet. 2019 Feb 11;27(5):691–700. doi: 10.1038/s41431-019-0351-3 (PMC6462008; doi:10.1038/s41431-019-0351-3)
Supplement: Supplementary file 1 — Supplementary materials [file 41431_2019_351_MOESM1_ESM.docx]

**Supplementary materials**

**Supplementary material 1. Measures used in this study**

1: Items discussed during counselling. We used self-constructed items to measure how important participants considered the items discussed by the GP on a Likert-scale from 1-5 (anchors very unimportant- very important) and how they rated the time spent on each of these topics on a Likert scale from 1-5 (anchors too little-too much).

In the GPs’ checklist, items 1-8 and 11 are the same. In addition to these, the following two items were included: The norms and values of the couple in relation to this ECS test and The turnaround time and communication of the test-result. We asked GPs to indicate to what extent they discussed these during the counselling (yes, somewhat, no).

| **1** | The purpose of ECS for severe genetic conditions. |
| --- | --- |
| **2** | Information about the conditions included in the test |
| **3** | Chances of being a carrier of one of the conditions included in the test |
| **4** | Chances of having a child with one of the conditions in the test |
| **5** | Test-procedures (both partners providing a blood sample) |
| **6** | Certainty of the test result |
| **7** | Costs associated with the test |
| **8** | Reproductive options in case of a positive result |
| **9** | Your norms and values in relation to this ECS test |
| **10** | Your partner’s norms and values in relation to this ECS test |
| **11** | Other aspects of a healthy pregnancy (folic acid, no smoking/alcohol) |

2: Patient satisfaction with counselling. We measured patient satisfaction with counselling via the 7-item Clinical Genetics Satisfaction Indicator (CGSI) adopted by the Dutch Society of Clinical Genetics(1). Internal consistency was high (Cronbach’s α=0.92) and comparable to that of the English version (Cronbach’s α=0.90)(1). We also included a self-constructed item to rate overall patient satisfaction with pre-test counselling on a scale from 1 -5 (*very unsatisfied-very satisfied*).

3: Degree of informed choice. The degree of informed choice was evaluated using an adapted version of the Multi-Dimensional Measure of Informed Choice (MMIC)(2). A choice was considered informed if participants had sufficient knowledge and made a decision consistent with their attitude towards participating in the ECS-test(2). Firstly, we constructed five knowledge items to cover the essentials of ECS:

1. A carrier of a severe genetic condition from this test has a change in the gene for this condition, but does not have the condition itself. (true)
2. If my partner and I will be told that we are not carriers of the same condition, then this means that we will have a healthy child. (False)
3. If my partner and I will be told that we are carriers of the same condition, we will have a 25% or 1 in 4 chance of having a child affected by the condition. (True)
4. If my partner and I will be told that we are carriers of the same condition, we can decide ourselves what we want to do with this information. (True)
5. A normal test result means that there is still a very small risk that we will have a child affected by one of the conditions in the test. (True)

Response mode was “true”, “false” or “I do not know” for each item. Moreover, attitude towards participating in the ECS test was measured with two items on a seven-point scale (good/bad; acceptable/unacceptable). These scores were reclassified as negative (1,2), neutral (3-5) and positive (6,7). We defined sufficient knowledge as a score ≥3/5 items answered correctly, which is in accordance with a recent study on informed choice in non-invasive prenatal testing for chromosomal abnormalities(3). Attitude was defined as positive if both items were scored positive. Attitude was defined as negative if both items were scored as negative. All other scores were classified as neutral. The attitude was considered in accordance with their decision if they either accepted testing and displayed a positive attitude, or did not accept testing and displayed a negative attitude.

**References**

1. Zellerino B, Milligan SA, Brooks R, Freedenberg DL, Collingridge DS, Williams MS. Development, testing, and validation of a patient satisfaction questionnaire for use in the clinical genetics setting. Am J Med Genet Part C Semin Med Genet. 2009;151C(3):191–9.

2. Marteau TM, Dormandy E, Michie S. A measure of informed choice. Heal Expect. 2001;4,:99

-108.

3. van Schendel R V., Page-Christiaens GCL, Beulen L, Bilardo CM, de Boer MA, Coumans ABC, et al. Trial by Dutch laboratories for evaluation of non-invasive prenatal testing. Part II—women’s perspectives. Prenat Diagn. 2016;36:1091–8.
